# The Effector Domain Region of the *Vibrio vulnificus* MARTX Toxin Confers Biphasic Epithelial Barrier Disruption and Is Essential for Systemic Spread from the Intestine

**DOI:** 10.1371/journal.ppat.1006119

**Published:** 2017-01-06

**Authors:** Hannah E. Gavin, Nike T. Beubier, Karla J. F. Satchell

**Affiliations:** 1 Department of Microbiology-Immunology, Northwestern University Feinberg School of Medicine, Chicago, IL, United States of America; 2 Department of Pathology, Northwestern University Feinberg School of Medicine and Northwestern Memorial Hospital, Chicago, IL, United States of America; University of Illinois, UNITED STATES

## Abstract

*Vibrio vulnificus* causes highly lethal bacterial infections in which the Multifunctional Autoprocessing Repeats-in-Toxins (MARTX) toxin product of the *rtxA1* gene is a key virulence factor. MARTX toxins are secreted proteins up to 5208 amino acids in size. Conserved MARTX N- and C-terminal repeat regions work in concert to form pores in eukaryotic cell membranes, through which the toxin’s central region of modular effector domains is translocated. Upon inositol hexakisphosphate-induced activation of the of the MARTX cysteine protease domain (CPD) in the eukaryotic cytosol, effector domains are released from the holotoxin by autoproteolytic activity. We previously reported that the native MARTX toxin effector domain repertoire is dispensable for epithelial cellular necrosis *in vitro*, but essential for cell rounding and apoptosis prior to necrotic cell death. Here we use an intragastric mouse model to demonstrate that the effector domain region is required for bacterial virulence during intragastric infection. The MARTX effector domain region is essential for bacterial dissemination from the intestine, but dissemination occurs in the absence of overt intestinal tissue pathology. We employ an *in vitro* model of *V*. *vulnificus* interaction with polarized colonic epithelial cells to show that the MARTX effector domain region induces rapid intestinal barrier dysfunction and increased paracellular permeability prior to onset of cell lysis. Together, these results negate the inherent assumption that observations of necrosis *in vitro* directly predict bacterial virulence, and indicate a paradigm shift in our conceptual understanding of MARTX toxin function during intestinal infection. Results implicate the MARTX effector domain region in mediating early bacterial dissemination from the intestine to distal organs–a key step in *V*. *vulnificus* foodborne pathogenesis–even before onset of overt intestinal pathology.

## Author Summary

The bacterium *Vibrio vulnificus* causes highly lethal infections in susceptible individuals exposed to contaminated water or seafood. *V*. *vulnificus* produces a MARTX toxin, which plays a significant role in bacterial virulence. The large MARTX toxin has numerous functional regions that have been characterized *in vitro*, but have not been functionally assessed during infection. In this study, we use a mouse model to show that a region of the MARTX toxin–the effector domain repertoire–is required for bacterial virulence. This region of the toxin promotes disruption of the intestinal epithelial barrier and movement of bacteria from the initial site of infection to the liver and spleen. Surprisingly, bacterial movement from the intestine occurs even in the absence of overt intestinal tissue damage, indicating that more subtle actions of the effector domains are responsible for promoting systemic bacterial spread. Together, our results advance the field of MARTX toxin research by functionally characterizing the toxin during infection. Results indicate that cytolytic effects on epithelial cells observed *in vitro* do not directly correspond to activities that are critical to bacterial pathogenesis *in vivo* and should encourage the study of diverse MARTX toxin functions.

## Introduction

The United States Department of Agriculture (USDA) reports that 1 in 6 Americans fall ill with a foodborne infection every year, resulting in more than 3000 fatalities and an estimated $15 billion in economic burden due to combined medical costs, productivity loss, and death. Of the cited foodborne illnesses, the most economically costly on a per-case basis are those caused by the Gram-negative, marine bacterial pathogen *Vibrio vulnificus* [1]. Since *V*. *vulnificus* can be concentrated out of the water by filter-feeders, such as oysters, susceptible individuals often contract gut infections from consumption of contaminated seafood [2, 3]. Within the intestine, the microbe is adept at transversing the intestinal barrier to spread systemically, at which point gastrointestinal symptoms are supplanted by bacterial sepsis [4] These intestinal infections can be severe resulting in hospitalization rates of more than 90% and fatality rates that exceed 50% [1, 5–10]. Although *V*. *vulnificus* cases remain rare, annual *V*. *vulnificus* case counts have increased over the past 15 years in the United States, and the infection is prevalent in endemic countries, including Japan, Taiwan, and South Korea [5, 11, 12]. Moreover, there is a growing risk of pathogen exposure in historically non-endemic areas as highlighted by case reports from countries including Sweden, Germany, France, and Denmark [13, 14]. Thus, the rise in *V*. *vulnificus* infection incidence, while in part attributable to improved reporting, correlates with increasing sea surface temperature and incidence of this disease is expected to increase with climate change [5, 15].

Pathogenic bacteria are known for secreting virulence factors that promote infection of host organisms. In the case of *V*. *vulnificus*, the *rtxA1* gene encodes a secreted multifunctional autoprocessing repeats-in-toxins (MARTX) toxin that is the dominant virulence factor for intestinal infection [16]. The 5206 a.a. MARTX toxin produced by *V*. *vulnificus* strain CMCP6 contains long regions of highly conserved tandem amino acid repeats at the N- and C-termini. These regions are required for toxin secretion, and formation of the MARTX toxin pore, which has been estimated at 1.63nm [17, 18]. Between the repeat regions are situated a cysteine protease domain (CPD) and a region of modularly organized effector domains [19]. The pore is thought to serve as the route for translocation of the central toxin region containing the CPD and effector domains across the eukaryotic plasma membrane from the extracellular space to the cytosol of target cells. Stimulated by the eukaryotic-specific molecule inositol hexakisphosphate, the CPD is activated to cleave after leucine residues located between effector domains, thereby releasing bacterial effector proteins into the cytosol of the targeted eukaryotic cell [20].

The *V*. *vulnificus* MARTX toxin is associated with numerous cytopathic and cytotoxic functions *in vitro*. Specifically, the toxin plays a role in lysis of numerous eukyarotic cell types [21, 22], cytoskeletal dysfunction as illustrated by epithelial cell rounding [18], inflammasome activation [23], inhibition of phagocytosis [24–26], and induction of apoptosis [27]. Moreover, the discrete effector domains present in the inner region of MARTX toxins are being biochemically characterized to discern their enzymatic functions. Effector domains from various MARTX toxins are now known to inhibit Rho GTPases [28, 29], cleave Ras and Rap1 [30, 31], inhibit autophagy [32], induce apoptosis [33], and crosslink actin [34–36] Yet it has been challenging to discern functional relationships between discrete portions of the MARTX toxin and *V*. *vulnificus* virulence.

We have previously demonstrated that the MARTX toxin effector domain region is dispensable for toxin secretion from the bacterium and toxin translocation to target cells, indicating that secretion and translocation functions are conferred by the conserved repeat regions [18]. In fact, the toxin repeat regions and the CPD comprise a sufficiently robust platform to deliver a heterologous beta-lactamase (Bla) in place of the native MARTX toxin effector repertoire [18, 28]. Notably, the MARTX toxin pore is also sufficient to induce necrotic cytotoxicity of HeLa cells *in vitro*, even in the absence of effector domains [17, 18, 22]. In light of the intestinal tissue necrosis observed during *V*. *vulnificus* intragastric (i.g.) infection, it might follow that the MARTX toxin repeat region pore, sufficient for cellular necrosis *in vitro*, would be sufficient for MARTX-associated intestinal damage, bacterial dissemination, and death as linked to colonization of the liver and spleen [9, 16, 37–39]. Yet, the effector domain region is retained across *V*. *vulnificus* isolates and is required for *in vitro* cell rounding and apoptosis induced by the bacterium [18, 33]. Therefore, we hypothesized that the MARTX toxin effector domain region is important for i.g. pathogenesis of *V*. *vulnificus*.

In this study, we characterize the functional roles of MARTX toxin regions in *V*. *vulnificus* pathogenesis and virulence during i.g. infection. We find that sum of the MARTX toxin effector domains induce rapid loss of transepithelial resistance and increased paracellular permeability *in vitro* prior to the induction of intestinal epithelial cell lysis. Moreover, MARTX effector domains are required for bacterial dissemination from the intestines to the liver and spleen very early during i.g. infection. Surprisingly, overt intestinal epithelial necrosis is not a prerequisite to bacterial dissemination. Together these data indicate that the focus of the MARTX toxin as a virulence factor should de-emphasize its lytic function and emphasize toxic mechanisms of delivered effector domains that confer *V*. *vulnificus* virulence potential.

## Results

### Bacterial strain selection and verification

**Fig 1:**
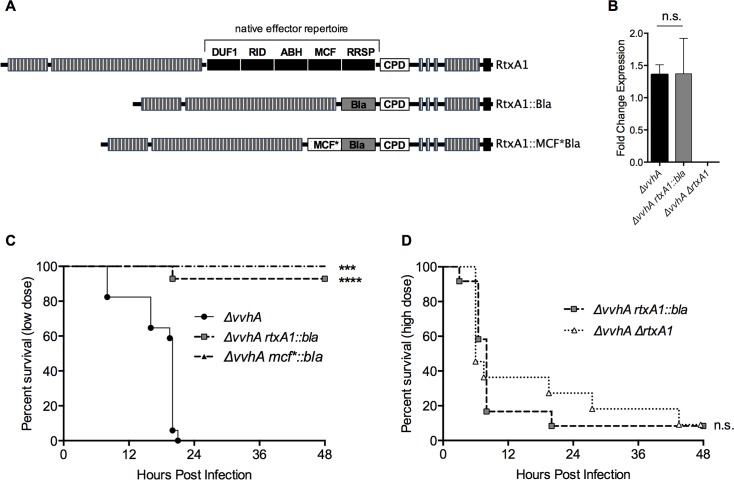
*V*. *vulnificus* MARTX toxin effector domains confer virulence *in vivo*. (A) Schematic representation of toxins produced by Δ*vvhA*, Δ*vvhA rtxA1*::*bla*, and ΔvvhA mcf*::*bla* strains derived from *V*. *vulnificus* CMCP6*rif*. DUF1 = domain of unknown function in the first position; RID = Rho Inactivation Domain; ABH = Alpha/Beta Hydrolase domain; MCF = Makes Caterpillars Floppy-like domain; RRSP = Ras/Rap1 Specific Protease domain; CPD = Cysteine Protease domain; striped boxes = repeat regions; (B) Mean ± standard deviation (s.d.) of native and modified *rtxA1* gene expression measured by qRT-PCR. (C-D) Mice infected i.g. with low dose (C, 2–5 x10^6^ CFU) or high dose (D, 5–8 x 10^8^ CFU) of the indicated strains were monitored over 48 hours for survival. Statistical significance determined by (B) Student’s t-test or (C,D) a log-rank test, **p<0.01, ***p<0.001, n.s. = not significant.

The MARTX cytotoxin product of *rtxA1* is established as a potent virulence factor of *V*. *vulnficus* infection [16, 25]. The VvhA cytolysin/hemolysin product of the *vvhA* gene is also cytolytic and functions additively to virulence, yet the MARTX toxin exerts the dominant effect [16]. To isolate MARTX-associated phenotypes from those of VvhA, we utilized a variant of *V*. *vulnificus* CMCP6 with an internal out-of-frame deletion in *vvhA* [18]. In this strain, the wild-type version of the MARTX toxin (RtxA1, Fig 1A) is produced.

The *rtxA1* locus has then been manipulated in this Δ*vvhA* background. The Δ*vvhA rtxA1*::*bla* strain was previously generated by replacing the gene sequence encoding for the MARTX toxin effector domains with an in-frame sequence encoding TEM1 Bla to produce the modified RtxA1::Bla toxin (Fig 1A). This effector-free strain expresses and secretes the modified Bla MARTX toxin, as previously characterized [18]. An *rtxA1* null strain was also previously generated via internal deletion, resulting in the Δ*vvhA ΔrtxA1* strain [18].

To verify that CMCP6 genetic manipulation did not compromise *rtxA1* gene expression, quantitative real-time PCR (qRT-PCR) was employed. Gene expression from mutant strains was compared to that of the CMCP6*rif* parent strain following growth in Luria Burtani (LB) broth. At the mRNA level, there was no detectable difference of gene expression of *rtxA1* between the Δ*vvhA* and Δ*vvhA rtxA1*::*bla* strains (Fig 1B). Therefore, *rtxA1* mRNA expression is not affected by the presence or absence of the gene sequence encoding effector domains.

### Effector domains are required for MARTX toxin-associated virulence

To examine the role of MARTX toxin effector domains in *V*. *vulnificus* virulence, mice were inoculated i.g. with either the parental *ΔvvhA* strain or the effector-free *ΔvvhA rtxA1*::*bla* strain (Fig 1C, low dose). The *ΔvvhA* strain caused fatality in 100% of mice by 24 hours post infection (hr p.i.). In contrast, the *rtxA1*::*bla* was lethal to just 7% of infected mice (Fig 1C). Thus, the *rtxA1*::*bla* strain is significantly attenuated, demonstrating that the MARTX toxin effector domains are instrumental to toxin-associated virulence.

To test for an association between *in vitro* cytolytic activity and virulence, mice were infected at a 100-fold higher dose with either *ΔvvhA rtxA1*::*bla–*which causes *rtxA1-*mediated lysis *in vitro*–or *ΔvvhA ΔrtxA1 –*which does not induce lysis *in* vitro. No significant differences in survival outcomes were observed between the two groups (Fig 1D). Notably, some *vvhA-* and *rtxA1-*independent virulence was observed at these high-doses. This could be due to bacterial components, such as LPS, and/or minor virulence factors, such as the VvpE metalloprotease, all of which may influence pathogenesis at very high dose in the absence of dominant virulence factors [40, 41]. Nonetheless, the *ΔvvhA rtxA1*::*bla* strain does not confer additional virulence over the null mutant [18].

Although the effector-free MARTX toxin expressed by *ΔvvhA rtxA1*::*bla* is sufficient to cause necrotic cytotoxicity *in vitro*, the kinetics of this process are delayed in the absence of MARTX toxin effector domains [18]. Therefore, slower cell lysis kinetics could contribute to the observed virulence attenuation of *ΔvvhA rtxA1*::*bla* compared to *ΔvvhA*. If this hypothesis were true, it should then follow that a strain with intermediate MARTX-dependent lysis kinetics would exhibit intermediate virulence.

To address this question, a recently described *rtxA1* variant with a catalytically-inactive MCF-like effector domain integrated into the *rtxA1*::*bla* gene to generate the modified toxin RtxA1::MCF*Bla (Fig 1A) [33] was used. In this strain, the presence of the effector stimulates the rate of *in vitro* cell lysis to a level intermediate to that of *ΔvvhA rtxA1*::*bla* and *ΔvvhA* [33]. However, the ΔvvhA mcf*::*bla* strain was completely attenuated during i.g. infection and showed no increased virulence above that of the *ΔvvhA rtxA1*::*bla* strain (Fig 1C). Despite exhibiting intermediate *rtxA1*-dependent lysis kinetics *in vitro*, this strain does not exhibit intermediate virulence phenotype. Therefore, lysis kinetics do not correlate to strain virulence potential.

Collectively, these studies indicate that the ability of a given *V*. *vulnificus* strain to induce lysis *in vitro* does not correlate with bacterial virulence for i.g. inoculated mice. Rather, MARTX effector domains delivered by the holotoxin, while not required for cell lysis, are essential for MARTX toxin-associated virulence.

### MARTX effector domains are required for bacterial dissemination

It is known that detection of bacteria in the liver and spleen correlates with lethality in mouse models of *V*. *vulnificus* infection [38, 39]. Clinically, outcomes of *V*. *vulnificus* human infection are considerably worse once the infection has become septic [6]. Therefore, bacterial dissemination from the initial site of the infection constitutes a key step in *V*. *vulnificus* pathogenesis. Moreover, the *rtxA1* gene has been linked to the dissemination process [16, 25].

To examine the role of MARTX toxin effector domains in *V*. *vulnificus* intestinal colonization and dissemination, we inoculated mice i.g. with *ΔvvhA*, *ΔvvhA rtxA1*::*bla*, or *ΔvvhA ΔrtxA1* at the same dose used to determine relative virulence of these strains. Knowing that initial lethality was observed approximately 8 hr p.i., we selected 6 hr p.i. to euthanize mice and collect organs such that all mice could be examined prior to death. Isolated organs were homogenized and the resulting homogenate plated to determine the bacterial load per organ.

**Fig 2:**
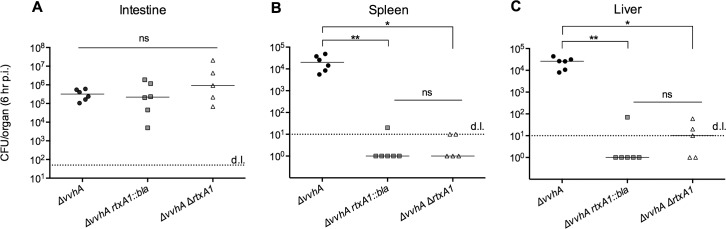
MARTX toxin effector domains are required for bacterial dissemination from intestine to distal organs. Live rifampin-resistant *V*. *vulnificus* recovered by plating of tissue homogenates of the (A) whole intestine, (B) spleen, and (C) liver at 6 hr p.i. for 5–6 mice per group. Plot symbols represent CFU/organ for individual mice, lines indicate mean, with statistical significance determined by one-way ANOVA followed by multiple comparison’s test to determine *p* values. *p<0.05, **p<0.01, ***p<0.001, n.s. = not significant, d.l. = detection limit.

No differences in bacterial recovery from the intestine were detected across mice infected with *ΔvvhA*, *ΔvvhA rtxA1*::*bla* and *ΔvvhA ΔrtxA1* (Fig 2A). Interestingly, this indicates that the bacterial load of the intestine at 6 hr p.i. is independent not only of the MARTX toxin effector domains, but also of the toxin in its entirety. Therefore, neither the MARTX toxin effector domain region nor the repeat regions influence intestinal bacterial load 6 hr p.i. Moreover, early intestinal colonization does not account for virulence differences among the inoculated strains.

Bacterial load in the liver and spleen of the same mice were also quantified at 6 hr p.i. (Fig 2B and 2C). While the *ΔvvhA* strain is detected in these organs at 2–5 x 10^4^ CFU/organ, neither the *ΔvvhA rtxA1*::*bla* nor the *ΔvvhA ΔrtxA1* strain disseminated to this level. In fact, no colonies grew from the majority of liver and spleen homogenates from *ΔvvhA rtxA1*::*bla* or *ΔvvhA ΔrtxA1-*infected mice even when plated at a detection limit of 10 CFU/organ.

The inability to detect *V*. *vulnificus* in distal organs at a meaningful level in the absence of the MARTX toxin effector domain region reinforces the integral role of the MARTX toxin in *V*. *vulnificus* pathogenesis. In addition, this result indicates that lytic action conferred by MARTX toxin repeat regions is not sufficient to facilitate bacterial transit to distal organs. Rather, an intact MARTX toxin effector domain region is required for bacteria to reach, and survive in, distal organs including the liver and spleen.

### Neither overt intestinal tissue damage nor epithelial apoptosis is a prerequisite to *V*. *vulnificus* dissemination

Bacterial dissemination from the intestine to distal organs necessitates bacterial transit across the protective intestinal epithelial barrier from intestinal lumen to bloodstream, and resistance to immune defense mechanisms, particularly phagocytosis, encountered at each of these locations. While the MARTX toxin is known to facilitate bacterial resistance to phagocytosis [25, 26, 28], equivalent bacterial loads were observed in the intestines of mice at 6 hr p.i. Therefore, at this time point, at least in the intestine, *V*. *vulnificus* are not differentially susceptible to immune clearance dependent upon *rtxA1*, though this occurs at later time points [16].

In previous studies, significant intestinal epithelial tissue damage has been observed in both mice and humans following i.g. *V*. *vulnificus* infection [9, 16, 37]. This damage has subsequently been attributed to the additive function of secreted exotoxins VvhA and MARTX [16]. To test the relationship between *ΔvvhA rtxA1*::*bla* lysis *in vitro*, intestinal epithelial damage during infection, and bacterial dissemination, mice were inoculated i.g. with the *ΔvvhA*, *ΔvvhA rtxA1*::*bla*, or *ΔvvhA ΔrtxA1* strains. At 6 hr p.i., the entire intestine was collected and analyzed with hemotoxalin and eosin (H/E) staining.

**Fig 3:**
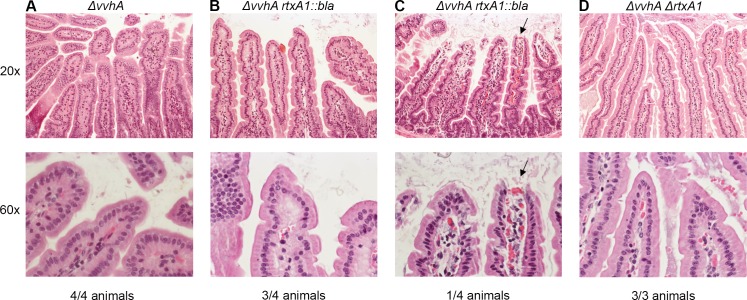
Intestinal tissue pathology is not a prerequisite to *V*. *vulnificus* dissemination. Histological cross sections at 20x (top row) and 60x (bottom row) from Swiss-rolled, H&E-stained small intestines of mice inoculated i.g. with 2–5 x 10^6^ CFU of bacterial strains as indicated at top.

Surprisingly, no significant pathology was observed in any intestinal tissues at 6 hr p.i. The epithelia remain intact in 4/4 *ΔΔvvhA*-infected mice, 3/4 *ΔvvhA rtxA1*::*bla*-infected mice, and 3/3 *ΔvvhA ΔΔrtxA1-*infected mice (Fig 3). In 1/4 *ΔvvhA rtxA1*::*bla-*infected mice, the intestine showed observable bacterial staining in the lumen and the small intestinal epithelium showed some damage at villous tips (Fig 3). This outlier sample demonstrates that rare events resulting in rapid bacterial expansion can lead to epithelial damage and supports a model in which bacterial outgrowth in the intestine at later infection time points (8–12 hr p.i.) indeed causes pathological changes, as previously shown [16]. However, this event was not representative. Therefore, we conclude that *V*. *vulnificus* does not induce extensive intestinal epithelial necrosis by 6 hr p.i. and the overt damage previously observed during later infection or in neutropenic mice is not solely responsible for dissemination [16, 37]. In fact, dissemination is initiated by the MARTX toxin effector domain region prior to the onset of fulminant intestinal tissue damage *in vivo*.

The absence of fulminant intestinal tissue pathology during i.g. *V*. *vulnificus* infection suggested that the bacterium might instead be inducing localized apoptosis of intestinal epithelial cells rather than lysis. Indeed, it has been reported that *V*. *vulnificus* can induce mitochondrial-mediated apoptosis in an *rtxA1*-dependent manner [27]. Apoptotic cells can be observed by an experienced pathologist in H&E stains tissue sections at high magnification. However, no major differences among tissue samples were observed in the examined H&E sections.

Nonetheless, this result was confirmed using an apoptosis-specific stain. The same embedded intestinal tissue used for H/E staining were also stained for cleaved caspase-3. Sporadic apoptosis is observed in the intestinal epithelial layer as indicated by positive staining for cleaved caspase-3 (S3 Fig). However, no differences were observed dependent upon *rtxA1*. Therefore, differences in apoptosis do not account for differences in bacterial dissemination among *V*. *vulnificus* strains and do not provide a mechanism by which epithelial breach is occurring.

### Bacterial dissemination does not induce organ pathology

**Fig 4:**
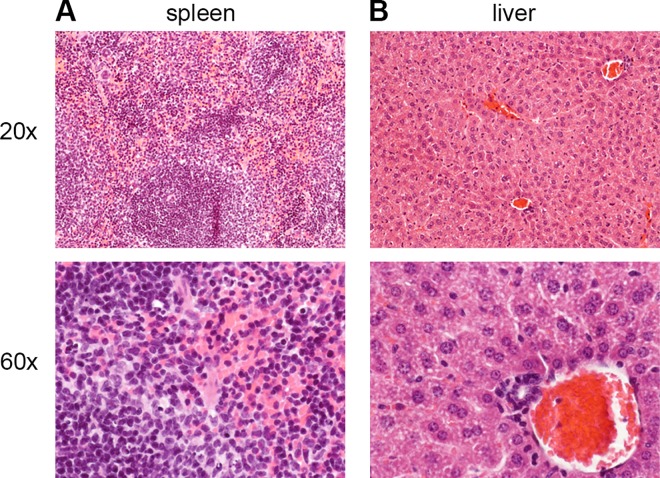
Disseminated bacteria do not induce organ pathology at 6 hr p.i. Histological cross sections at 20x (top row) and 60x (bottom row) from H&E-stained (A) spleen and (B) liver of mice inoculated i.g. with 5 x 10^6^ CFU of *ΔvvhA* strain.

Previous studies have linked *V*. *vulnificus* dissemination from the intestine to the liver and spleen with lethal infection outcomes [38, 39]. To assess organ damage, the spleen and liver were isolated from mice infected with *ΔvvhA*, *ΔvvhA rtxA1*::*bla*, or *ΔvvhA ΔrtxA1* at 6 hr p.i. and assessed by histological examination. Despite the presence of >10^4^ CFU *ΔvvhA* in the spleen and liver of infected mice (Fig 2), these organs retain their normal morphology at 6 hr p.i. and tissues do not exhibit overt pathology (Fig 4). Therefore, disseminated *V*. *vulnificus ΔvvhA* do not induce direct spleen and liver organ damage at 6 hr p.i, despite the rapid onset of animal mortality beginning at 8 hr p.i. Absent overt pathology in the form of either tissue necrosis or apoptosis, we reasoned that the changes induced by *V*. *vulnificus* MARTX to facilitate bacterial dissemination must be subtler and not dependent on gross effects observable by pathology.

### The MARTX toxin rapidly disrupts transepithelial resistance *in vitro*

**Fig 5:**
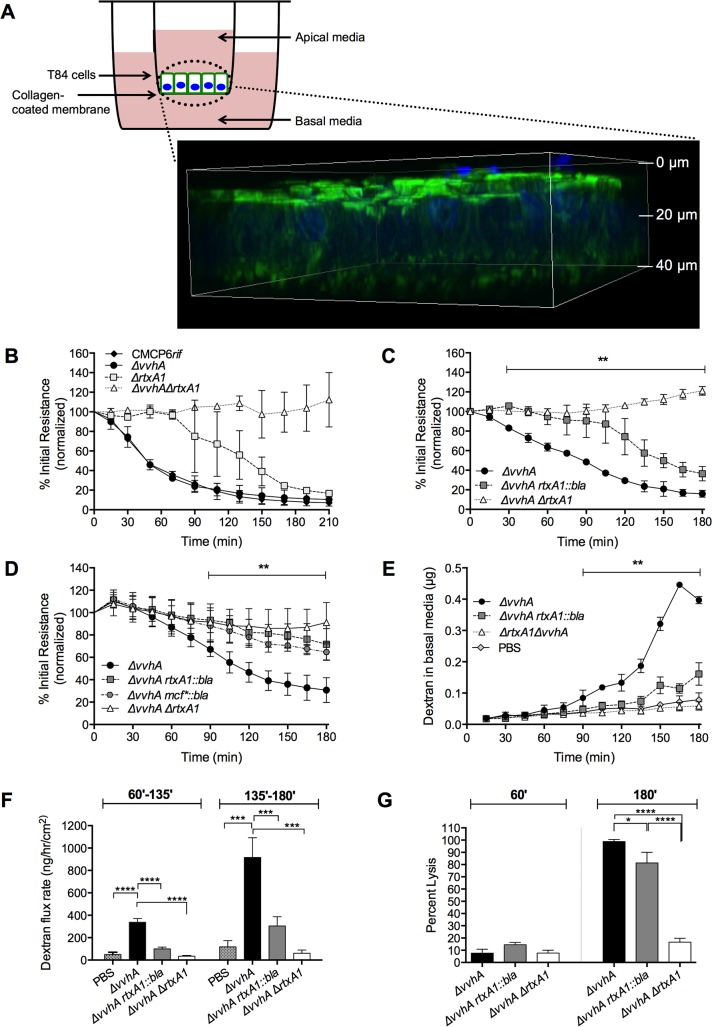
MARTX toxin effector domains rapidly induce intestinal barrier dysfunction *in vitro*. (A) Schematic representation and representative 3-D confocal microscopy image of untreated T84 monolayer stained for nuclei (DAPI, blue) and actin (phalloidin AlexaFluor 488, green). (B-D) TER of polarized monolayers monitored over 180–210 minutes following apical exposure to the indicated strains of *V*. *vulnificus* at MOI = 2.6 (B-C) or MOI = 0.26 (D). Data are expressed as mean percent initial resistance ± s.d. relative to PBS-exposed monolayers. (E) Mean (μg) ± s.d. amount of fluorescently labeled, 3-kD dextran in basal media of polarized T84 monolayers following exposure to the indicated strains at MOI = 2.6. (F) Biphasic dextran flux rates observed during *V*. *vulnificus* exposure, derived from the slope of the dextran curves displayed in panel (E), from 60–135 minutes (left) or 135–180 minutes (right). (G) Lysis of polarized T84 monolayers, relative to samples treated with Triton X-100, at 60 minutes (left) and 180 minutes (right). For (C-E), data were analyzed by two-way ANOVA with statistically significant differences, as marked, compared for *ΔvvhA* to *ΔvvhA rtxA1*::*bla* (C-E) or ΔvvhA mcf*::*bla* (D) across all the indicated time points, **p<0.01. For (F,G), data were analyzed by one-way ANOVA followed by a multiple comparison’s test to determine *p* values; *p<0.05, ***p<0.001, ****p<0.0001.

Previous work that predominantly focused *V*. *vulnificus* research on cell lysis extensively uses lactate dehydrogenase (LDH) release from nonconfluent, adherent epithelial cell monolayers as an *in vitro* system for MARTX-dependent cytotoxicity [16–18, 22, 24]. For a more relevant three-dimensional culture model to monitor intestinal epithelial barrier breach events *in vitro*, the interaction between *V*. *vulnificus* and polarized confluent T84 colonic epithelial cells was studied (Fig 5A). T84 cells were cultured as monolayers in transwells to transepithelial resistance (TER) of ≥1000 Ω*cm^2^. Log phase bacteria were added to the apical surface to mimic luminal i.g. bacterial exposure. Based upon preliminary dosing experiments (S1 Fig) and bacterial ratios relevant to *in vivo* infection (S1 Text), subsequent experiments were conducted at multiplicity of infection (MOI) 2.5 or 0.25.

Initial experiments examined the individual contributions of *vvhA* and *rtxA1* to characterize interactions between the epithelial monolayer and bacterial exotoxins *in vitro* (Fig 5B). When applied to the apical surface of T84 monolayers, CMCP6*rif* rapidly induced intestinal barrier dysfunction as demonstrated by a 50% decrease from initial TER after 60 minutes and more than 80% drop over 210 minutes. T84 monolayers exposed to *V*. *vulnificus ΔvvhA* exhibited a drop in TER identical to monolayers exposed to CMCP6*rif*. In contrast, T84 monolayers exposed to *ΔrtxA1* retained initial TER to nearly 90 minutes, exhibiting a significant delay and attenuation of TER disruption relative to CMCP6*rif* and *ΔvvhA*-exposed monolayers. When monolayers were exposed to *ΔvvhA ΔrtxA1*, TER was maintained over the course of the experiment to the endpoint at 210 minutes and, over multiple experiments, often resulted in a rise in TER. Together these results indicate that both the VvhA hemolysin and MARTX toxin are sufficient for TER drop over a 3-hour time scale, but only the MARTX toxin accounts for the rapid loss of TER initiated shortly after addition of bacteria. Further, the contribution of VvhA that occurs after 120 min is not essential, additive or synergistic (Fig 5B).

### MARTX toxin effector domains are essential for rapid loss of T84 monolayer TER

Having established the importance of the MARTX holotoxin in TER loss, the role of MARTX toxin regions was examined (Fig 5C). T84 monolayers were co-incubated with *V*. *vulnificus ΔvvhA*, *ΔvvhA rtxA1*::*bla*, or *ΔvvhA ΔrtxA1*. Monolayers exposed to *ΔvvhA* dropped to 50% resistance in 60–75 minutes of co-incubation, as previously observed. However, the integrity of the monolayers exposed to *ΔvvhA rtxA1*::*bla* was maintained to approximately 100 minutes. Therefore, compared to the *ΔvvhA* strain, the *ΔvvhA rtxA1*::*bla* strain is significantly delayed in its ability to disrupt T84 monolayer integrity. T84 monolayers exposed to *ΔvvhA rtxA1*::*bla* for more than 100 minutes gradually exhibited a drop in TER, though TER loss induced by the *ΔvvhA rtxA1*::*bla* strain was attenuated throughout the duration of the experiment compared to that induced by *ΔvvhA*. Monolayers exposed to *ΔvvhA rtxA1*::*bla* strain were also distinct from the *ΔvvhA ΔrtxA1*-exposed monolayers, as the double mutant did not experience any disruption of TER. Thus, MARTX effector domains are necessary for rapid-onset monolayer disruption, but pore formation by the MARTX repeat regions is sufficient for T84 disruption past 100 minutes of co-incubation.

To test whether different toxin translocation and lysis kinetics previously observed in HeLa cells influenced bacterial interactions with T84 monolayers, the ΔvvhA mcf*::*bla* strain was again employed (Fig 5D) [33]. With the goal of observing even subtle differences between the strains, the applied dose was reduced 10-fold to MOI 0.25. However, even at this lower dose there was no detectable difference between the ΔvvhA mcf*::*bla* and *ΔvvhA rtxA1*::*bla*-exposed T84 monolayers. Both strains remained drastically attenuated in the ability to disrupt T84 TER compared to *ΔvvhA*. The *ΔvvhA* strain induced 50% TER loss by approximately 100 minutes–a delay relative to the 10-fold higher dose, but an increase relative to either of the strains lacking active MARTX effector domain regions. At the same 100-minute time point, monolayers exposed to ΔvvhA mcf*::*bla* or *ΔvvhA rtxA1*::*bla* retained approximately 90% initial resistance.

**Fig 6:**
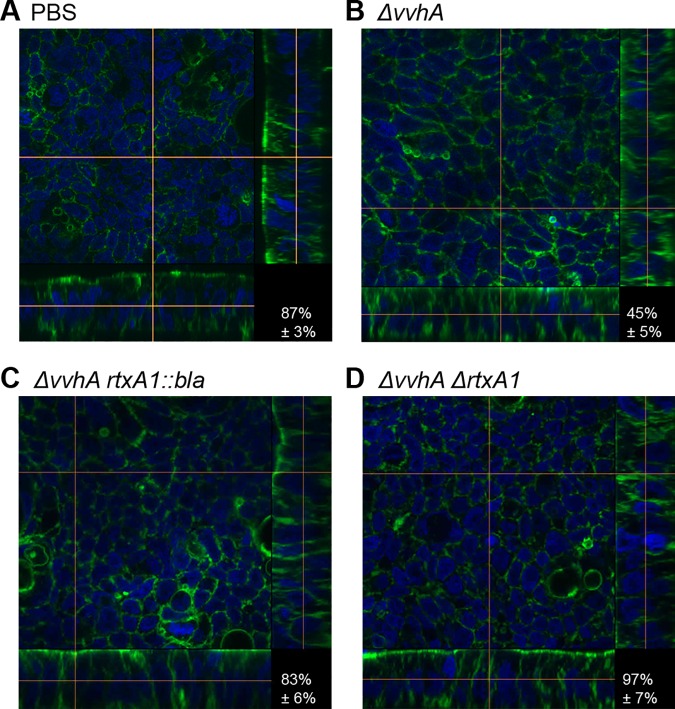
Cytoskeletal morphology is maintained during early barrier dysfunction at 60 mins. Representative confocal microscopy cross-section and *z*-stack images of T84 monolayers stained for nuclei (DAPI, blue) and actin (phalloidin AlexaFluor 488, green) following exposure to (A) PBS (negative control); (B) *ΔvvhA;* (C) *ΔvvhA rtxA1*::*bla;* or (D) *ΔvvhA ΔrtxA1*. Percent initial resistance reported as mean ± s.d. for three monolayers per strain is indicated in the lower right corner of each panel.

Surprisingly, this loss of TER by 60 mins was not due to overt actin depolymerization (Fig 6). Indeed, despite dropping to 45% initial resistance, the *ΔvvhA*-exposed monolayer exhibits actin morphology akin to the PBS control monolayers. Specifically, all samples retained characteristic honeycomb-like actin morphology in the *x-y* plane and columnar cellular structure in the monolayer *z*-plane. Therefore, the rapid initial loss of TER upon addition of *V*. *vulnificus* to T84 monolayers is not an artifact of differences in lysis kinetics of the wild-type RtxA1 toxin compared to RtxA1::Bla and is not due to extensive loss of actin structure.

### The MARTX toxin induces a biphasic disruption of paracellular epithelial permeability

A common mechanism by which the integrity of a monolayer can be compromised is disruption of cell-cell junctions that results in increased paracellular permeability. To examine mechanisms of MARTX-induced barrier dysfunction, paracellular permeability to small molecules was examined with use of a fluorescently tagged, 3-kD dextran. The molecule cannot pass through cells and is likewise typically excluded from passing between cells by tight junctions. However, when intercellular junctions are disrupted, the dextran molecule gains passage between cells. Following application of dextran to the apical transwell chamber, PBS or bacteria were added to monolayers and basal media was sampled for dextran transit over time.

Appreciably greater amounts of dextran were sampled from the basal media of *ΔvvhA*-exposed monolayers compared to monolayers exposed to *ΔvvhA rtxA1*::*bla*, or *ΔvvhA ΔrtxA1* (Fig 5E). Neither PBS mock-exposed nor *ΔvvhA ΔrtxA1*-exposed monolayers allowed more than 0.09 μg of dextran to cross from the apical to the basal compartment of the T84 transwells over the tested 180 minute timecourse. Similarly, a maximum of 0.15 μg of dextran was detected in *ΔvvhA rtxA1*::*bla-*exposed monolayers. However, the *ΔvvhA*-exposed monolayers allowed passage of 0.45 +/- 0.01 μg of fluorescent dextran (Fig 5E).

In *ΔvvhA*-exposed transwells, an upward trend in basal dextran levels began between 45 and 60 minutes. By 90 minutes, significant differences in dextran transit between the *ΔvvhA* monolayers and all other monolayers were evident (Fig 5E). In contrast, only basal levels were observed in *ΔvvhA rtxA1*::*bla* and *ΔvvhA ΔrtxA1*-exposed monolayers prior to 135 minutes. From 135–180 minutes, increased levels of dextran were sampled from the basal media of monolayers exposed to *ΔvvhA rtxA1*::*bla* compared to *ΔvvhA ΔrtxA1* or PBS-exposed monolayers, though these amounts were still considerably less than the basal dextran sampled from *ΔvvhA-*exposed monolayers.

These *in vitro* monolayer experiments revealed that both TER disruption and dextran flux exhibit biphasic characteristics. Specifically, the *ΔvvhA* strain caused rapid decay of TER, while *ΔrtxA1* (Fig 5B) and *ΔvvhA rtxA1*::*bla* (Fig 5C) caused only late onset TER disruption. The *ΔvvhA* strain rapidly induced paracellular permeability, but the rate of dextran flux between 135 and 180 minutes (900 ng/hr/cm^2^) was significantly greater than the flux rate from 60–135 minutes (350 ng/hr/cm^2^) (Fig 5F). In *ΔvvhA rtxA1*::*bla-*exposed monolayers, dextran flux rates were significantly greater in the 135–180 minute time frame (300 ng/hr/cm^2^) compared to 60–135 minutes (100 ng/hr/cm^2^). Overall, it was concluded that the MARTX toxin, when interacting with a polarized columnar monolayer, exerts two MARTX-dependent mechanisms of barrier disruption: one shortly after addition of bacteria and mediated by the effector domains and one later after addition of bacteria linked to the repeat regions.

### Late, but not early, onset TER disruption coincides with cell lysis

Since the repeat regions are known to be sufficient for lysis of unpolarized cells, the contributions of *V*. *vulnificus* MARTX toxin regions to T84 cell lysis was explored as a mechanism for late vs early onset loss of TER. Monolayers were exposed to *ΔvvhA*, *ΔvvhA rtxA1*::*bla*, or *ΔvvhA ΔrtxA1*. Sixty minutes following bacterial application, resistance of the *ΔvvhA-*exposed monolayers dropped to 50% initial while *ΔvvhA rtxA1*::*bla*, or *ΔvvhA ΔrtxA1* monolayers retained TER >90%, as observed in previous independent experiments (Fig 5). Monolayer cell lysis was measured by sampling LDH release to media in both the apical and basal transwell chambers and expressed relative to LDH release from monolayers treated with 0.1% Triton X-100. LDH release to the basal transwell chamber was never detected (S2 Fig) so monolayer lysis was quantified using media sampled from the apical chamber. The absence of LDH in the basolateral chamber may also explain the absence of bacteria in the same compartment, if the lower edge of the monolayer and appendages filling the filter pores do not provide a clear path across a partially lysed monolayer (S2 Fig). Despite the large drop in TER in *ΔvvhA*-treated monolayers by 60 minutes, *ΔvvhA* cell lysis at 60 minutes averaged less than 10% and was no greater than the low levels likewise observed in PBS, *ΔvvhA rtxA1*::*bla*, or *ΔvvhA ΔrtxA1*-exposed monolayers (Fig 5G, 60 minutes). Therefore, rapid MARTX-dependent loss of TER by *ΔvvhA* occurs independent of cell lysis.

By contrast, at 180 minutes following bacterial application, *ΔvvhA*-exposed monolayers retained just 10% initial resistance, *ΔvvhA rtxA1*::*bla*-exposed monolayers exhibited 50% initial resistance, and *ΔvvhA ΔrtxA1*-exposed monolayer resistance had increased to 180% initial. At this late timepoint, *ΔvvhA ΔrtxA1* lysis levels remained <20% while both *ΔvvhA* and *ΔvvhA rtxA1*::*bla* induced lysis exceeding 80% of cells in the monolayer. Therefore, T84 cell lysis occurred after prolonged monolayer exposure and corresponds to increased dextran flux and TER loss in both *ΔvvhA* and *ΔvvhA rtxA1*::*bla*-exposed monolayers.

### Generation of a library of validated *Δeffector* strains in CMCP6*rif rtxA1*

**Fig 7:**
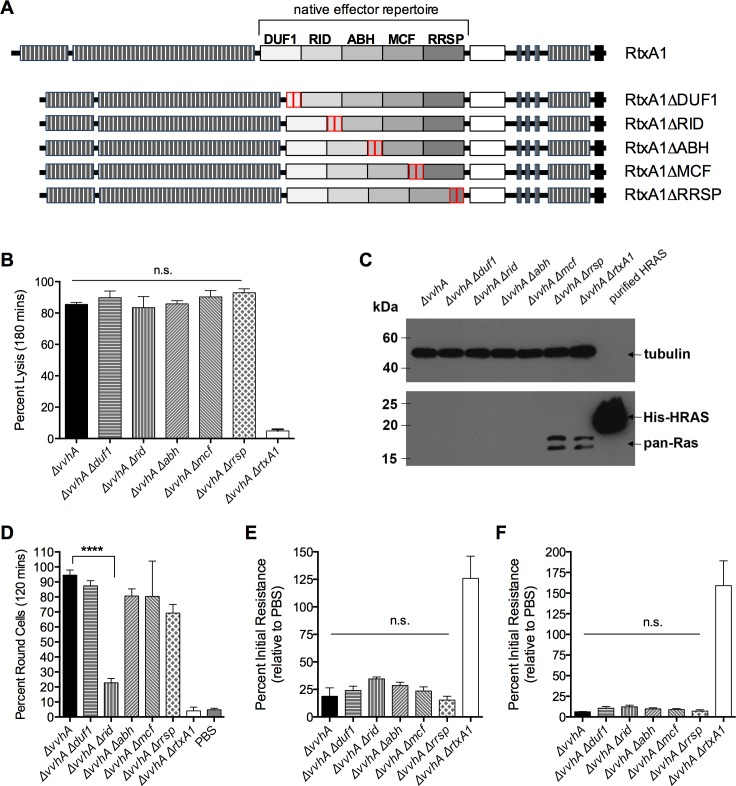
Roles of individual MARTX toxin effector domains. (A) Schematic representation of the *Δeffector* strain library. DUF1 = domain of unknown function in the first position; RID = Rho Inactivation Domain; ABH = Alpha/Beta Hydrolase domain; MCF = Makes Caterpillars Floppy-like domain; RRSP = Ras/Rap1 Specific Protease domain; CPD = Cysteine Protease domain; striped boxes = repeat regions; (B) Lysis of T84 monolayers exposed to the indicated bacterial strains for 180 minutes, expressed relative to Triton X-100 treated monolayers. (C) Western blot using lysates from HeLa cells exposed to the indicated strains for 60 minutes at MOI = 100, probed with anti-Ras10 antibody (bottom) and anti-tubulin (top) (D) Percent of rounded HeLa cells observed upon exposure to the indicated bacterial strains for 120 minutes at MOI = 10. (E-F) TER of polarized monolayers measured at 60 mins (E) or 120 mins (F) minutes following apical exposure to the indicated strains of *V*. *vulnificus* at MOI = 2.6. Data analyzed by one-way ANOVA followed by multiple comparison’s test to determine *p* valules; ****p<0.0001, n.s. = not significant.

Now knowing the important role of the MARTX effector domain repertoire in its entirety, the role of individual domains within the region was explored. Ideally, this experiment would have been conducted using strains with point mutations in the active sites of each MARTX effector domain so as to generate catalytically inactive effector domains in the context of the MARTX holotoxin. However, the catalytic residues of numerous domains have not as yet been identified, and some catalytic point mutants are known to exert intermediate effects when target-binding activity is retained [29, 33, 42, 43]. Therefore, a library of strains was generated in the *ΔvvhA* background in which each strain harbors an in-frame deletion in the *rtxA1* coding region to eliminate a single effector domain from the otherwise functional toxin (Fig 7A).

All *Δeffector* strains were first validated as inducing release of LDH from polarized T84 monolayers at 180 minutes (Fig 7B), demonstrating that the modified *rtxA1* genes expressed toxin that retained the cell lysis activity linked to the repeat regions. In addition, *Δduf1*, *Δrid*, *Δabh*, and *Δmcf* were confirmed to induce loss of detectable Ras from HeLa cells when co-incubated with the target cells for 1 hour at MOI = 100 (Fig 7C). Ras was detected in cells incubated with the *Δrrsp* strain as expected due to loss of the RRSP-dependent cleavage of Ras [30].

Cytopathic epithelial cell rounding in response to *V*. *vulnificus* has also been previously attributed to the MARTX effector domain repertoire [18]. As shown in Fig 7D, a strain expressing the wild-type MARTX toxin induced rounding of more than 90% of HeLa cells in 120 minutes (*ΔvvhA*, Fig 7D) while a *ΔvvhAΔrtxA1* strain does not induce any effect above the background. The *Δduf1*, *Δabh*, *Δmcf*, and *Δrrsp* strains similarly induced cell rounding, independently validating these strains are producing functional MARTX toxins. As expected, due to the disruption of its Rho-inactivated domain linked to cytoskeleton disassembly, epithelial cell rounding was significantly reduced in HeLa cells incubated with the *Δrid* strain (Fig 7D).

Thus, all *Δ*effector strains were validated as retaining the ability to lyse cells and retain or lose activity specifically linked to at least two of the effector domains.

### No single MARTX toxin effector domain is essential for the rapid loss of T84 monolayer TER

These validated strains were next used to define the effector domains essential for rapid loss of TER in polarized T84 monolayers. For this experiment, polarized T84 monolayers were exposed apically to *Δeffector* strains and TER was measured at 60 minutes, when early loss of TER is most pronounced, and at 120 minutes, when effector-independent barrier dysfunction begins (Fig 5). Interestingly, all of the *Δeffector* strains induced loss of TER at levels comparable to the strain expressing the entire effector domain repertoire (Fig 7E and 7F). Therefore, no single MARTX effector is essential for induction of epithelial barrier dysfunction *in vitro*. In addition, this result indicates that despite its requirement for induction of cell rounding in nonconfluent HeLa epithelial cells, Rho inactivation induced by RID does not alone account for TER loss at 60 minutes. This suggests that MARTX effectors function redundantly or synergistically with regard to epithelial barrier disruption.

## Discussion

The important role of the MARTX toxin product of the *rtxA1* gene as a virulence factor during *V*. *vulnificus* infection has now been appreciated for nearly a decade [44]. This potent cytotoxin has been linked to induction of multiple forms of cell death *in vitro* [17, 22, 27]. During i.g. infection, fulminant intestinal tissue damage has been observed. Moreover, bacterial dissemination and sepsis are phenotypes intimately linked to lethal infection outcomes [6, 37–39, 45]. Together, these data have led to a prevailing model that massive toxin-mediated destruction of the intestinal epithelial barrier is the key mechanism by which *V*. *vulnificus* exits the intestine culminating in lethal sepsis. The data presented here indicate a paradigm shift in our conceptual understanding of early *V*. *vulnificus* transmigration of the intestinal epithelial barrier. Indeed, the cytopathic activities of the MARTX toxin initiate dissemination earlier than previously appreciated and the early mechanisms involved are far subtler in nature.

In studying the contribution of MARTX toxin regions to holotoxin function, we previously identified the MARTX repeat regions as sufficient for formation of pores in the eukaryotic plasma membrane resulting in epithelial cellular necrosis [18]. These bacteria that produce a MARTX toxin able to lyse cells, but absent the effector domain region, were indistinguishable during intestinal infection of mice from bacteria that did not produce the toxin at all (Fig 1). Thus, it is the entire toxin, inclusive of the effector domains, that confers MARTX-mediated virulence during i.g. *V*. *vulnificus* infection. Notably, genetic and biochemical functional characterization of MARTX toxin complexity previously lent hypothetical support to this result. Yet, these data represent the first direct experimental evidence that non-lytic functions previously attributed to the MARTX toxin (such as cytoskeleton disassembly, induction of apoptosis, inhibition of autophagy, and modulation of stress signaling [27, 30, 32, 33, 46, 47]) must play an important role in pathogenesis.

To further understand how the toxin contributes to virulence, a physiologically relevant *in vitro* model system using polarized T84 cells was newly optimized for use with *V*. *vulnificus*. These studies demonstrated that the MARTX toxin induces biphasic intestinal epithelial dysfunction in the form of early-onset increases in paracellular permeability, followed by late-onset cell lysis. The effector domain region of the MARTX toxin is responsible for the rapid loss of barrier function. Yet, studies with bacteria that express toxins lacking individual effector domains reveal that no single effector domain is essential for this rapid intestinal epithelial disruption. Rather there must be an additive or synergistic function from multiple effector domains in loss of epithelial barrier function, a mechanism by which effector domains may contribute to bacterial translocation from the intestine to the liver and spleen following i.g. infection in mice, resulting in *V*. *vulnificus* sepsis.

The dissociation between MARTX-associated virulence, epithelial barrier breach, and lysis is further reflected in results from study of animal histopathology. The MARTX holotoxin does not induce overt intestinal tissue damage or excess apoptosis during early infection. This finding is consistent with the T84 experiments where rapid loss of TER was not linked to dramatic loss of cytoskeleton structure. Paracellular permeability increase in the absence of major changes to cytoskeletal morphology suggests that more delicate modulation of cytoskeletal dynamics, such as those at intercellular junctions, is occurring.

By contrast, the second phase of TER loss was linked to cell damage resulting in release of LDH. This second event likely accounts for the outlier pathology sample that showed observable bacterial staining in the intestinal lumen as well as epithelial damage at villous tips suggest that rare stochastic events leading to early-stage bacterial outgrowth facilitate epithelial damage. However, the absence of significant necrotic or apoptotic phenotypes in all *ΔvvhA-*infected mice indicates that the MARTX effector domain region is necessary for dissemination not because it causes or induces overt tissue damage, but because it induces early bacterial transit from the intestine via other mechanisms.

It was recently observed that the *V*. *vulnificus* elastase product of *vvpE* modulates paracellular permeability by altering tight junction protein dynamics [41]. However here no significant dysfunction is observed in monolayers exposed to *ΔvvhA ΔrtxA1*, indicating that *vvpE* does not increase permeability or intestinal damage in this context. The apparent discrepancy is likely due to differences both in study methods and bacterial MOI. *vvpE* was shown to increase intestinal permeability *in vivo* when mice were inoculated i.g. with a high dose of 1.1x10^9^ CFU. However, the *in vitro* MOI used in the present study corresponds to intestinal bacterial loads following inoculation with 5x10^6^ CFU, a nearly 200-fold lower dose. Interestingly, observed *rtxA1* and *vvhA-*independent virulence at high-dose (Fig 1D) does indicate a role for either bacterial components, such as LPS, or for minor virulence factors, in the absence of the two major cytotoxins. These combined data suggest that *vvpE* may play a functional role at the intestinal epithelial barrier, and in virulence, at high bacterial load.

A consideration now is how the bacteria are moving across the barrier *in vivo*. Modulation of paracellular permeability by the MARTX effector domain region may act to directly facilitate paracellular bacterial transit between epithelial enterocytes for subsequent transport to the lymphatics or blood stream in the intestinal lamina propria. Further, the same mechanisms by which the MARTX effector domain repertoire dysregulates enterocytes may also dysregulate other specialized cell populations in the intestinal epithelial monolayer. M cells, responsible for luminal antigen sampling, are present in the epithelial layer that covers lymphoid nodules and Peyer’s patches. The so-called “weak point of the intestinal epithelial barrier” [48], M cells are known to provide a route for transepithelial migration of viable bacteria, including *V*. *cholerae*, from the intestinal lumen to the underlying Peyer’s patches [49, 50]. While M cell luminal sampling is integral to proper antigen presentation and immune responsiveness, pathogens such as *Salmonella*, *Shigella*, and *Yersinia* exploit the properties of M cells to access the mucosa and spread systemically [50, 51]. Goblet cells have been implicated in transcytosis of *Listeria* and thus represent another putative route by which *V*. *vulnificus* is breaching the intestinal barrier [52], potentially facilitating systemic bacterial spread.

An important caveat of this study is that events in the intestine are focused only during the early phase of infection, at 6 hr p.i., in the mouse. At this time point, the number of bacteria in the gut does not yet depend upon *rtxA1*, and toxin-mediated epithelial destruction has not yet occurred. Nonetheless, previous studies have demonstrated that these *rtxA1-*mediated phenomena certainly occur at later time points [16, 37]. This indicates that when known effects on *rtxA1-*dependent immune clearance [24, 25, 28] are not yet impacting intestinal bacterial load, strains producing the MARTX holotoxin are already detectable in the spleen and liver. It is postulated that within the bloodstream and at tissue sites, *rtxA1* and its various regions may also play a critical role in resistance to immune clearance promoting bacterial outgrowth at these sites. In human cases where *V*. *vulnificus* infection progresses to septic shock and multi-organ failure, this rise in bacterial loads in the liver and spleen may predict dissemination-associated fatality. However, direct damage to the spleen or liver by the bacteria may not be essential for death, given that mice exhibit no organ damage at 6 hr p.i. yet begin to die by 8 hr p.i.

**Fig 8:**
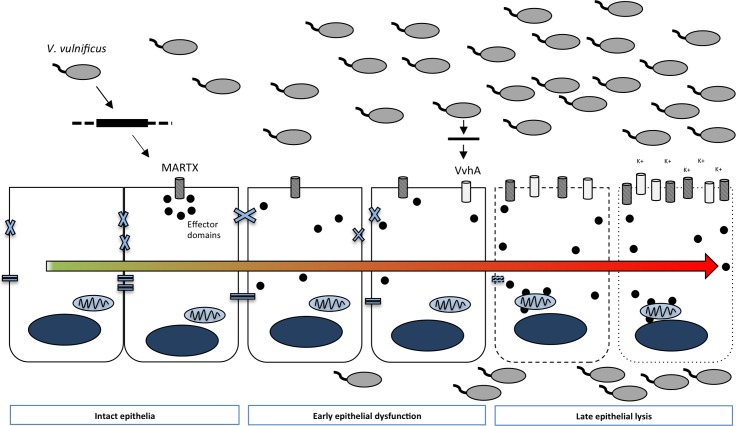
Summary model of MARTX effector-mediated intestinal barrier disruption. Epithelial monolayers exposed to *V*. *vulnificus* experience rapid monolayer dysfunction mediated by the MARTX toxin effector domain region. Monolayer dysfunction is characterized by increased paracellular permeability allows for bacterial passage across the normally protective epithelial monolayer. MARTX-mediated barrier dysfunction facilitates early bacterial dissemination from the intestinal lumen to distal organs, prior to the onset of overt intestinal tissue damage. As intestinal bacterial burdens increase, lysis-mediated barrier dysfunction is mediated by pore-forming toxins and does not require the MARTX effector domain region *in vitro*. Notably, however, early barrier dysfunction events mediated by the MARTX toxin effector domain region are required for bacterial virulence potential conferred by the MARTX toxin.

Overall, the results presented in this study support a model in which *V*. *vulnificus* bacteria expressing the MARTX holotoxin rapidly induce intestinal epithelial dysfunction in the form of increased paracellular permeability and transmigration (Fig 8). These early steps are sufficient to facilitate bacterial dissemination and associated virulence potential. Our evidence suggests that initial translocation of bacteria out of the intestine is mediated by the MARTX effector domains and occurs in the absence of overt tissue damage in the intestine, spleen and liver tissues. Subsequent bacterial outgrowth–or a sporatic event resulting in higher bacterial burden–then leads to intestinal tissue necrosis *in vivo* (Fig 8). However, in the absence of MARTX effector domain functions, the early breach of the barrier and early arrival of bacteria at distal organs does not occur, resulting in dramatically reduced virulence potential.

## Materials and Methods

### Bacterial strains and media

*V*. *vulnificus* strains *ΔvvhA*, *ΔvvhA rtxA1*::*bla*, *ΔvvhA mcf*::*bla*, and *ΔvvhA ΔrtxA1* were generated from the Korean clinical isolate *V*. *vulnificus* CMCP6*rif* as described previously [18, 33]. *Escherichia* coli strains DH5 αλ*pir*, SM10λ*pir*, and S17λ*pir* [53, 54] were used for new strain construction. Bacteria were routinely grown in Luria-Burtani (LB) broth (10 g tryptone, 5 g yeast extract, 5 g NaCl) containing 50 μg/mL rifampin or 10 μg/ml chloramphenicol as needed. For all experiments, *V*. *vulnificus* was streaked from frozen glycerol stocks onto LB plates. The following day, single colonies were grown in 2 mL LB broth overnight at 30^°^C and then subcultured 1:100 into LB without antibiotic and grown to mid-log phase. Cultures were pelleted and resuspended in sterile phosphate buffered saline (PBS, 10mM sodium phosphate, 140 mM NaCl, pH 7.4) to indicated concentrations based on optical density (A_600_).

### Generation of *Δeffector V*. *vulnificus* strains

The CMCP6 MARTX toxin CPD processing sites have not been precisely mapped although the boundaries of each effector domain have been defined based on extensive sequence alignment [19]. To ensure that processing of neighboring effectors was not negatively impacted by the deletion, the predicted processing sites were preserved along with 15% of the effector domain itself. The designed deletions correspond to the following nucleotides based on the CMCP6 sequence of Kim *et al*., [57] (National Center for Biotechnology Information Reference #NC_004460.2): *Δduf1 (Δ*5890–6699); *Δrid (Δ*6814–8688); *Δabh (Δ*8794–9279); *Δmcf* (*Δ*9490–10,722); and *Δrrsp* (*Δ*10,753–12,252).

Fragments corresponding to regions upstream and downstream of the desired deletion were either commercially synthesized (Integrated DNA Technologies, Coralville, IA) or amplified from the CMCP6 genome. The two fragments corresponding to each strain were assembled into digested pDS132 [55] either using Gibson Assembly according to the manufacturer’s protocols (New England Biolabs, Ipswich, MA) or by standard ligation using T4 DNA ligase. The resulting plasmids were confirmed by sequencing and transformed to SM10λ*pir* [53], and S17λ*pir* [54]. The Δ*effector* deletion plasmids were transferred to *V*. *vulnificus ΔvvhA* by conjugation followed by selection for double homologous recombination using sucrose counterselection to isolate recombinants as previously described [56]. Deletions in the *rtxA1* gene were confirmed by amplification of DNA across the deletion junction. Strains were validated by assessment of LDH release as described below, by western blotting for Ras proteolysis using the pan-Ras RAS10 (EMD Millipore, 05–516, 1:500) and tubulin (Sigma-Alrich, T6074, (1:10,000) monoclonal antibodies as previously described [30], and by assessment and quantification of HeLa cell rounding as previously described [18].

### qRT-PCR

Bacteria were grown as described above. RNA was extracted using Qiagen RNeasy Kit (Qiagen, 74104) and RNA Protect Bacteria Reagent (Qiagen, 76506) according to the manufacturer’s instructions. Isolated RNA was quantified using a Nano-Drop spectrophotometer. RNA was DNase treated using Turbo DNA Free Kit (Life Tech, AM1907). RNA was reversed transcribed using random hexamers (Roche, 11034731001) and Superscript III Reverse Transcriptase (Life Tech 18080–093) in the presence of RNasin (Promega N2611). qRT-PCR was carried out using iQ SYBR Green Supermix (BioRad 170–8880) and the BioRad iQ5 Multicolor RealTime PCR Detection System. Efficiency testing established primer efficiency of 83% and 87% for *rtxA1* and *16s rRNA* primer pairs, respectively. Primers used were: qRT-RTXF (5’AATACCGCTCTTCACAACC3’); qRT-RTXR (5’GCTTTCTGGGTGCTTACC3’); qRT-16srRNA_F (5’CTTGACATCCAGAGAATCTA3’); qRT-16srRNA_R (5’GACTTAACCCAACATTTCAC 3’)

Three separate qRT-PCR analyses were performed and data pooled following analysis. The *16s rRNA* gene served as internal housekeeping control. Fold change was calculated relative to parental CMCP6*rif*.

### Ethics statement

This study was carried out in strict accordance with the recommendations in the United States Public Health Service (USPHS) regulations and applicable federal and local laws. The protocol (Protocol No. IS00000905) was approved by the Northwestern University Institutional Animal Care and Use Committee (IACUC) as detailed in methods. All efforts were made to minimize suffering.

### Mouse i.g. infection

Female ICR mice were obtained from Charles River at age 32–38 days. Mice were anesthetized via intraperitoneal injection with 100 μL of anaesthetic cocktail containing 60–70 μg/kg ketamine and 12–14 μg/kg xylazine in PBS. Mice were inoculated i.g. using a 1-cm animal feeding needle attached to a 1-mL syringe. Mice were administered 50 μL of 8.5% aqueous sodium bicarbonate, followed immediately by 50 μL of bacterial culture containing the CFU indicated for a given experiment. Mice were monitored every 2 hours for the first 28 hours of the experiment and subsequently every 4–8 hours for a total experimental duration of 48 hr p.i. For low dose survival experiments, mice were inoculated with *V*. *vulnificus* strains *ΔvvhA* (*n* = 17), *ΔvvhA rtxA1*::*bla* (*n* = 14) and ΔvvhA mcf*::*bla* (*n* = 5) For high dose survival experiments, mice were inoculated with *ΔvvhA rtxA1*::*bla* (*n* = 12) and *ΔvvhA ΔrtxA1* (*n* = 11).

For bacterial recovery from organs, 5–6 mice per group were inoculated and then euthanized 6 hr p.i. The whole intestine (less the cecum) was excised and homogenized in 5 mL PBS. The liver and spleen were excised and each homogenized in 1 mL PBS. CFU/organ was calculated by plating serially diluted homogenates to LB agar containing rifampin to select for *V*. *vulnificus*.

For histopathology, 3–4 mice per group were inoculated and euthanized 6 hr p.i. The liver and spleen were dissected. A 1-cm sample was isolated from the proximal end of each segment of the small and large intestine (duodenum, jejunum, ileum, colon) for cross-sectional sampling. The remaining portions of each segment were opened along the longitudinal axis, rolled from proximal to distal, and sectioned to obtain samples in a “swiss roll” orientation. After 24–48 hours fixation in 10%-buffered formalin, all tissues were paraffin-embedded, processed, and stained with H&E. For immunohistochemistry, 4 μm of the same embedded tissues were sectioned, mounted on slides, and stained for apoptotic marker cleaved caspase-3 using the CP229C antibody from Biocare Medical, Concord, CA. All pathology sides were viewed and scored by N.T.B. blinded to treatment groups.

### T84 cell studies

T84 cells obtained from American Type Culture Collection (#CCL-248) were routinely grown in T84 media (1:1 DMEM/F12 Nutrient Mix (Gibco 11320–033)) supplemented with 10% fetal bovine serum (FBS) and 1% penicillin-streptomycin) to no more than 30 passages. For cell polarization, Costar Transwell Permeable Supports (6.5mm insert, 24-well plate, 3.0 μm polycarbonate membrane, Reference #3415) were coated with collagen and dried in a laminar flow hood overnight. Transwells were incubated with T84 media for 1 hour prior to the addition of 10^6^ T84 cells to the apical chamber of the transwell. Media was changed every 2–3 days for 10–14 days until monolayers reached ≥1000Ω/cm^2^ [58] as measured using an EVOM (World Precision Instruments). A minimum of three monolayers were prepared per assay condition.

One hour prior to bacterial co-incubation, monolayers were washed twice with warm Hanks Balanced Salt Solution (HBSS, Sigma-Aldrich) and media was replaced with 1:1 phenol-red free T84 media without FBS or antibiotic. Ten μL of PBS or the appropriate concentration of bacteria were applied drop-wise to the apical media. Monolayers were maintained at 37^°^C using a plate warmer. TER was measured in 15–20 minute intervals or as indicated in legends.

For confocal imaging, following 60 minutes of bacterial co-incubation, cells were fixed in 4% paraformaldehyde for 20 minutes. Monolayers were permeabalized using 0.1% TritonX-100. Actin was stained using AlexaFluor 488 phalloidin (ThermoFisher A12379) and nuclei were stained with 4′-6′-diamidino-2-phenylindole (DAPI, Life D1306), each according to manufacturer’s recommendations. Entire monolayers affixed to membrane were excised, mounted in Pro-Long Gold Antifade (Life Technologies, P36930) under a cover slip, and imaged using a Nikon A1R Spectral microscope.

For dextran flux studies, 200 μg fluorescein dextran (3-kD, ThermoFisher D3305) was added to the apical chamber immediately following application of PBS or bacteria to the apical chamber of transwells. Dextran transit across the monolayer was measured by sampling 20 μL media from the basal transwell chamber, after which the extracted volume was replaced with 20 μL fresh media. Sample fluorescence was measured using a Tecan Safire 2 fluorescence plate reader (excitation: 494 nm, emission: 521 nm) and amount of dextran (in pg) was calculated against a standard curve, accounting for volume differences due to sampling. Flux rates were reported as μg dextran/hr/cm^2^ from the slope of the plotted linear curve.

For LDH release assays, 100 μL of media was extracted from either the apical (Fig 5) or basal (S2 Fig) chambers of PBS, bacterial, or Triton X-100 incubated monolayers. One μL 100 mg/ml gentamicin was added and samples were centrifuged at 15,000*xg* for 1 minute. 50 μL of the resulting supernatant was transferred to a 96-well culture plate. LDH activity was measured using the Promega CytoTox Non-Radioactive Cytotoxicity Assay kit according to manufacturer’s instructions. The apical and basal samples were processed separately with data reported adjusting for volume.

### Statistical analyses

Statistical analyses were performed as indicated in figure legends using GraphPad Prism 6.0 software.

## Supporting Information

S1 Fig *V*. *vulnificus* CMCP6 induced barrier dysfunction at MOI 0.25. T84 monolayers (2/dose) exposed to CMCP6*rif* over the indicated MOI range. Data represent mean percent initial resistance ± s.d.
(TIFF)

S2 Fig Neither LDH nor bacteria are detected in the basal media of polarized T84 monolayers following bacterial exposure. Percent lysis as measured by LDH release to the basal media of polarized T84 monolayers at (A) 60 minutes or (B) 180 minutes. (C) Quantification of bacterial transmigration across transwell chamber membranes coated with collagen (marked *ΔvvhA* only) or collagen plus T84 cells. All data are reported as mean ± s.d.
(TIFF)

S3 Fig Epithelial apoptosis does not account for differences in bacterial dissemination. Embedded tissue slices stained for apoptosis marker cleaved caspase-3. No differences are observed among mice infected with the indicated strains. Apoptotic cells in the epithelium and the lamina propria are indicated by arrowheads at 40X.
(TIF)

S1 Text Details of calculation to establish MOI for T84 cell experiments. (PDF)
