# Supplementary figures and images for: The Effector Domain Region of the Vibrio vulnificus MARTX Toxin Confers Biphasic Epithelial Barrier Disruption and Is Essential for Systemic Spread from the Intestine
Source: PLoS Pathog. 2017 Jan 6;13(1):e1006119. doi: 10.1371/journal.ppat.1006119 (PMC5218395; doi:10.1371/journal.ppat.1006119)

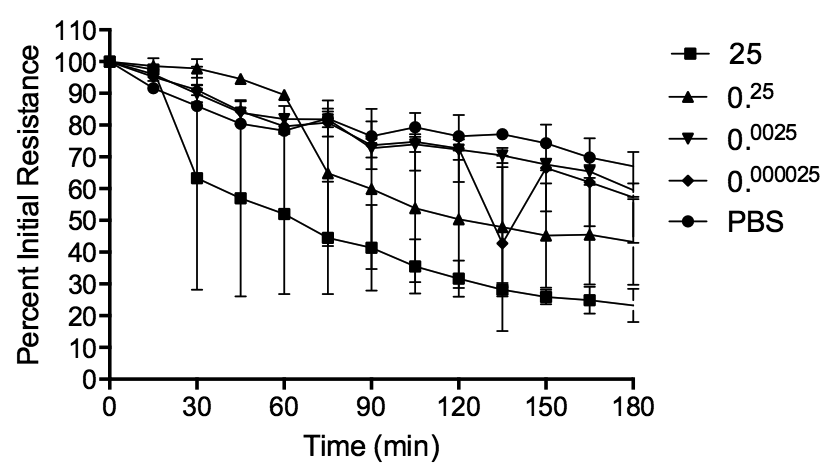

Supplement: S1 Fig — T84 monolayers (2/dose) exposed to CMCP6rif over the indicated MOI range. Data represent mean percent initial resistance ± s.d. (TIFF) [file ppat.1006119.s001.tiff]

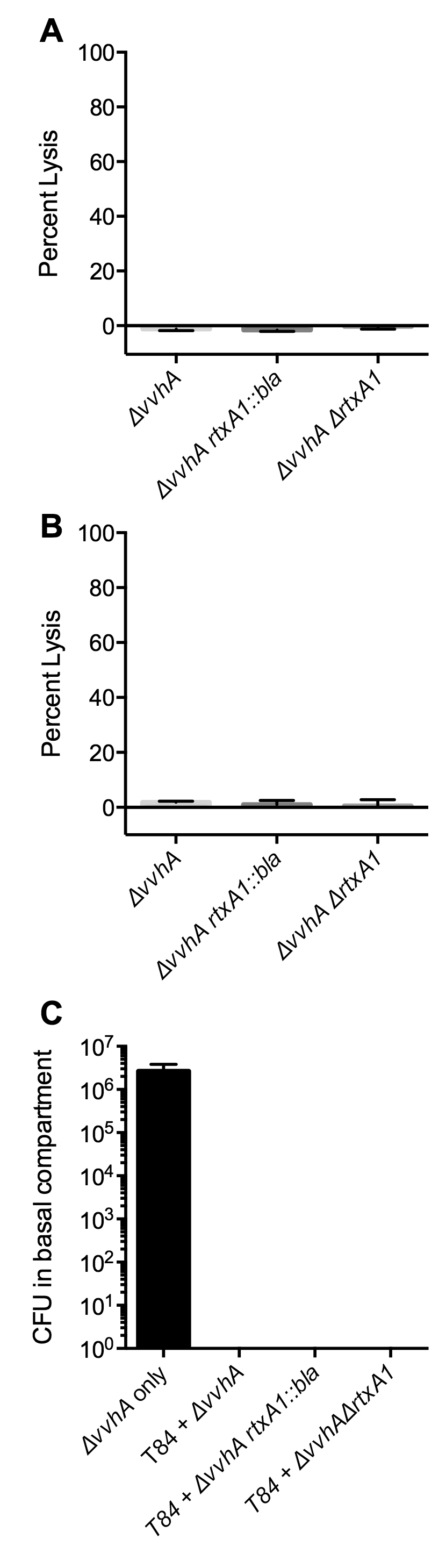

Supplement: S2 Fig — Percent lysis as measured by LDH release to the basal media of polarized T84 monolayers at (A) 60 minutes or (B) 180 minutes. (C) Quantification of bacterial transmigration across transwell chamber membranes coated with collagen (marked ΔvvhA only) or collagen plus T84 cells. All data are reported as mean ± s.d. (TIFF) [file ppat.1006119.s002.tiff]

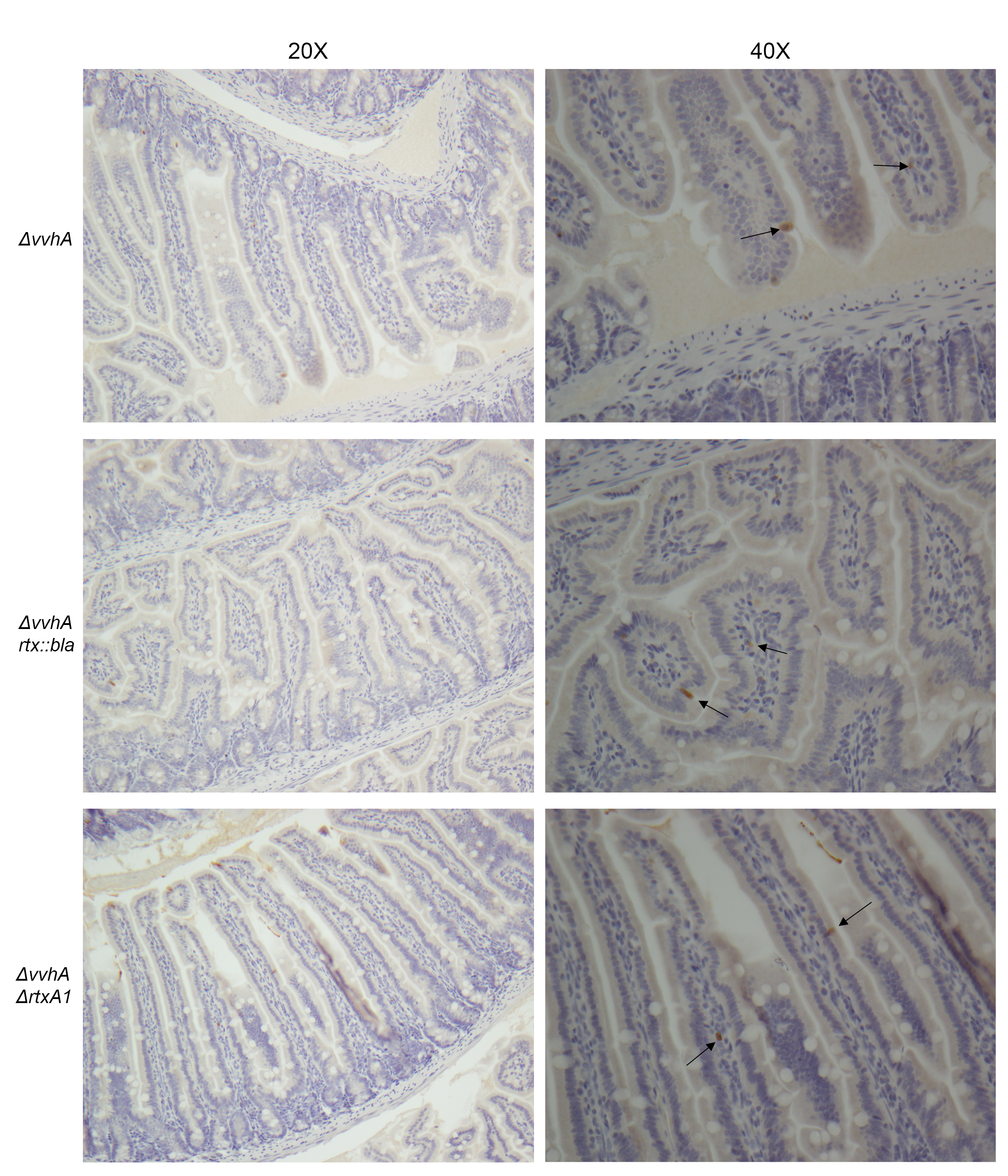

Supplement: S3 Fig — Embedded tissue slices stained for apoptosis marker cleaved caspase-3. No differences are observed among mice infected with the indicated strains. Apoptotic cells in the epithelium and the lamina propria are indicated by arrowheads at 40X. (TIF) [file ppat.1006119.s003.tif]
