# Supplementary material for: The Effector Domain Region of the Vibrio vulnificus MARTX Toxin Confers Biphasic Epithelial Barrier Disruption and Is Essential for Systemic Spread from the Intestine
Source: PLoS Pathog. 2017 Jan 6;13(1):e1006119. doi: 10.1371/journal.ppat.1006119 (PMC5218395; doi:10.1371/journal.ppat.1006119)
Supplement: S1 Text — (PDF) [file ppat.1006119.s004.pdf]

Supporting Text:

To determine relevant *V. vulnificus* exposure load for T84 monolayers *in vivo* multiplicity of infection (MOI) during i.g. mouse infection was estimated using mouse morphological characteristics, experimentally determined bacterial burdens, and empirical testing.

Calculation A: MOI based upon 8 hr p.i. bacterial burden in the mouse small intestine

Average mouse of 35-45 grams has, in the jejunum [47]:

$$Cell\ density: 1.90 * 10^7 \frac{cells}{cm^2}$$

Based upon the average of 4 and 6-week old age groups outlined in [48], the 32-38 day mice used in the current study (average 5-week) mice in this study should have an external intestinal epithelial surface area of:

$$Surface\ area = 21 \frac{cm^2}{small\ intestine}$$

Therefore, the total number of cells in the small intestinal epithelium is:

$$\#\ cells = cell\ density * surface\ area$$

$$= (1.90 * 10^7 \frac{cells}{cm^2}) * 22\ cm^2$$

$$= 40 * 10^7\ cells$$

According to unpublished lab data from a previous study [18], the small intestinal bacterial burden of a mouse lethally infected with *V. vulnificus ΔvvhA* at 8 hr. p.i. is approximately:

$$Bacterial\ burden: 5 * 10^9\ CFU$$

To estimate small intestinal MOI *in vivo:*

$$MOI = bacterial\ burden/\#\ of\ cells$$

$$= (5 * 10^9\ CFU)/(40 * 10^7\ cells)$$

$$= 12.5 \frac{CFU}{cell}$$

Calculation B: MOI based upon 6 hr p.i. bacterial burden in the whole intestine

Notably, this study examines bacterial burdens in the whole intestine, so the cells in the colon must be taken into account:

Surface area of the colon [48]:

$$Surface\ area = 7.6 \frac{cm^2}{colon}$$

Epithelial cell density of colon [47]:

$$Cell\ density = 8.98 * 10^6 \frac{cells}{cm^2}$$

Therefore, the total number of cells in the colon is:

$$\#\ cells = cell\ density * surface\ area$$

$$= (8.98 * 10^6 \frac{cells}{cm^2}) * 7.6\ cm^2$$

$$= 6.8 * 10^7\ cells$$

Total number of cells in the whole intestine:

$$Total\ cell\ \# = cell\ \#_{small\ intestine} + cell\ \#_{colon}$$

$$= (42 * 10^7 cells) + (6.8 * 10^7 cells)$$

$$= 49 * 10^7 cells$$

Moreover, bacterial burden in this study is examined at an earlier time point:

Bacterial burden in the intestine at 6 hr p.i.:

$$Bacterial\ burden = 5 * 10^5\ CFU$$

Thus the estimated MOI of whole intestine based on these colonization numbers is:

$$MOI = bacterial\ burden / \#\ of\ cell$$

$$= \frac{5 * 10^5\ CFU}{49 * 10^7\ cells}$$

$$= 0.001 \frac{CFU}{cell}$$

To calculate MOI *in vitro,* the number of cells in each monolayer was determined, using trypsin/ETDA to dissociate cells from a mature monolayer for counting. Subsequently, the MOI of a range of bacterial doses was determined:

T84 MOI at 10^1 CFU:

$$\frac{1 * 10^1\ CFU}{3.8 * 10^5 \pm 0.5\ cells} = 0.000026\ \frac{CFU}{cell} or\ 1\ CFU/38{,}461\ cells$$

T84 MOI at 10^7 CFU:

$$\frac{(1 * 10^7\ CFU)}{3.8 * 10^5 \pm 0.5\ cells} = 26 \frac{CFU}{cell}$$

This MOI range was then utilized for preliminary T84 dosing experiments (Supplementary Figure 1). Noting that T84 dysfunction was induced *in vitro* at MOI=0.26, subsequent experiments were carried out at either MOI=0.26 or MOI=2.6, both within the reasonable calculated range for putative *in vitro* MOI. Notably, localized differences in bacterial burden likely occur *in vivo* in the context of actual intestinal infection. Nonetheless, on average the numbers utilized in this study are supported by experimental data as demonstrated above. Moreover, the phenotypes noted in this study are observed at doses 4-40-fold lower than previous studies using MOI in the 10-100 range.
